# The effect of homozygous deletion of the *BBOX1* and Fibin genes on carnitine level and acyl carnitine profile

**DOI:** 10.1186/1471-2350-15-75

**Published:** 2014-07-01

**Authors:** Ali Rashidi-Nezhad, Saeed Talebi, Homeira Saebnouri, Seyed Mohammad Akrami, Alexandre Reymond

**Affiliations:** 1 Center for Integrative Genomics, University of Lausanne, 1015 Lausanne, Switzerland; 2 Department of Medical Genetics, School of Medicine, Tehran University of Medical Sciences, Poursina St, P.O. Box: 14155–6447, Tehran, Iran; 3 Maternal, Fetal and Neonatal Research Center, Tehran University of Medical Sciences, Tehran, Iran

**Keywords:** Carnitine, BBOX1, Fibin, CNV, Primrose syndrome

## Abstract

**Background:**

Carnitine is a key molecule in energy metabolism that helps transport activated fatty acids into the mitochondria. Its homeostasis is achieved through oral intake, renal reabsorption and *de novo* biosynthesis. Unlike dietary intake and renal reabsorption, the importance of *de novo* biosynthesis pathway in carnitine homeostasis remains unclear, due to lack of animal models and description of a single patient defective in this pathway.

**Case presentation:**

We identified by array comparative genomic hybridization a 42 months-old girl homozygote for a 221 Kb interstitial deletions at 11p14.2, that overlaps the genes encoding Fibin and butyrobetaine-gamma 2-oxoglutarate dioxygenase 1 (BBOX1), an enzyme essential for the biosynthesis of carnitine *de novo*. She presented microcephaly, speech delay, growth retardation and minor facial anomalies. The levels of almost all evaluated metabolites were normal. Her serum level of free carnitine was at the lower limit of the reference range, while her acylcarnitine to free carnitine ratio was normal.

**Conclusions:**

We present an individual with a completely defective carnitine *de novo* biosynthesis. This condition results in mildly decreased free carnitine level, but not in clinical manifestations characteristic of carnitine deficiency disorders, suggesting that dietary carnitine intake and renal reabsorption are sufficient to carnitine homeostasis. Our results also demonstrate that haploinsufficiency of *BBOX1* and/or *Fibin* is not associated with Primrose syndrome as previously suggested.

## Background

Carnitine (L-3-hydroxy-4-N,N,N-trimethylaminobutyrate) is crucial for energy metabolism. It is a conditional essential nutrient found in animals, numerous microorganisms and plants [1-3]. It allows the transport of activated fatty acids from the cytosol to the mitochondria, where they are beta-oxidized. Other functions of carnitine include peroxisome fatty acid oxidation, modulating intracellular coenzyme A homeostasis and removal of excess acyl groups from the body via the preferential renal excretion of acylcarnitines [4-7].

In mammals, carnitine homeostasis is achieved and maintained by a combination of absorption from dietary sources, *de novo* biosynthesis and efficient, renal tubular reabsorption [4,8]. Diet is the primary source of carnitine and dietary bioavailability of L-carnitine can vary considerably because of the broad range of nutritional choice. Meat, fish and dairy products are main sources in human, so vegetarians obtain very low amount of carnitine from their diet. However, compensatory mechanisms, including renal reabsorption in conjugation with *de novo* biosynthesis, are proficient in conserving carnitine homeostasis when dietary L-carnitine consumption is low [4,8,9].

L-carnitine is synthesized from the amino acid precursors lysine and methionine via four enzymatic reactions. These enzymes are found in all the human cells, with the exception of butyrobetaine-gamma 2-oxoglutarate dioxygenase 1(BBOX1) that is only expressed in the liver, kidneys and brain [5,10]. In contrast to the diet and renal reabsorption, the significance of carnitine *de novo* biosynthesis for energy homeostasis remains unclear [8], as no animal model and a single patient were described. While this patient was deficient for trimethyllysine hydroxylase epsilon (TMLHE), the first enzyme of the carnitine biosynthesis pathway [11,12], we report on the first instance of homozygous deletion of BBOX1, the last enzyme of that pathway.

## Case presentation

**Figure 1:**
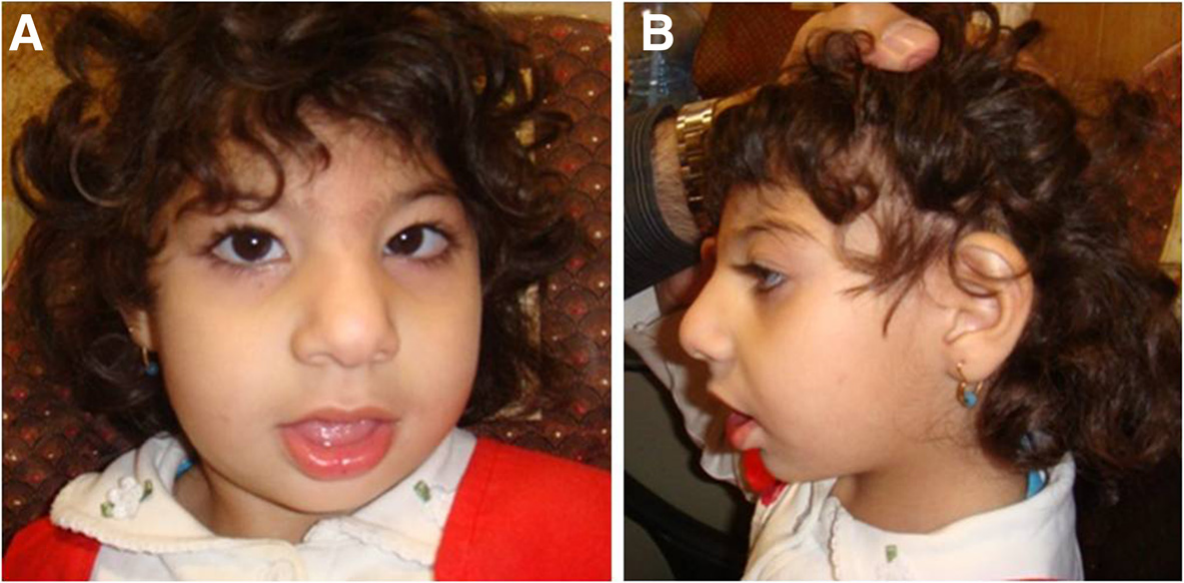
Proband’s facial (A) and ear (B) appearance.

The proband girl was born at 38 weeks of gestation to a 23 years old mother after an uneventful pregnancy. According to birth measurements, she suffered from Intra uterine growth restriction (IUGR) with disproportionate congenital microcephaly (Table 1). The parents are first-degree cousins. They have a second healthy child boy and did not experience any abortion or child death. Investigation of the family tree identified the spontaneous abortion of a first-degree cousin and a girl, 6^th^ degree proband’s relative, presented hypotonia, mental retardation and facial dysmorphism. At 42 months, the proband presented a mild dysmorphic face with short forehead, high nasal bridge and flat nasal root, down slant pulpebral fissure, epicanthal folds, mild strabismus, long eye lashes, retrognathia and large ears relative to her face (Figure 1A-B). Her anthropometric measurements showed growth retardation (Table 1). She was hyperactive with a history of drooling and several attacks of unprovoked seizures that are now under control without antiepileptic drug. Her neurological examination showed a mild generalized hypertonia with mild contracture in knees but her balance, motor, sensory and reflexes were all in the normal ranges. The proband, has a history of motor delay and spasticity in lower limbs, which was improved with physical therapy. On the contrary her speech delay showed no improvement upon therapy. The proband social and cognition axes of development were almost normal. The auditory brainstem response (ABR) was also normal. However, the brain magnetic resonance imaging (MRI) showed severe microcephaly and small frontal lobes with no evidence of gross structural abnormality.

**Table 1:** Proband’s anthropometric measurements

**Physical characteristics**	**Birth**	**Z-score**	**42 mths**	**Z-score**
**Weight**	2.4 kg	-1.99	9.5 kg	-3.58
**Height**	44 cm	-2.76	80 cm	-4.85
**Head circumference**	29 cm	-4.12	37.5 cm	-8
**Body Mass Index (BMI)**	12.40	-0.79	15.1	-0.15

**Figure 2:**
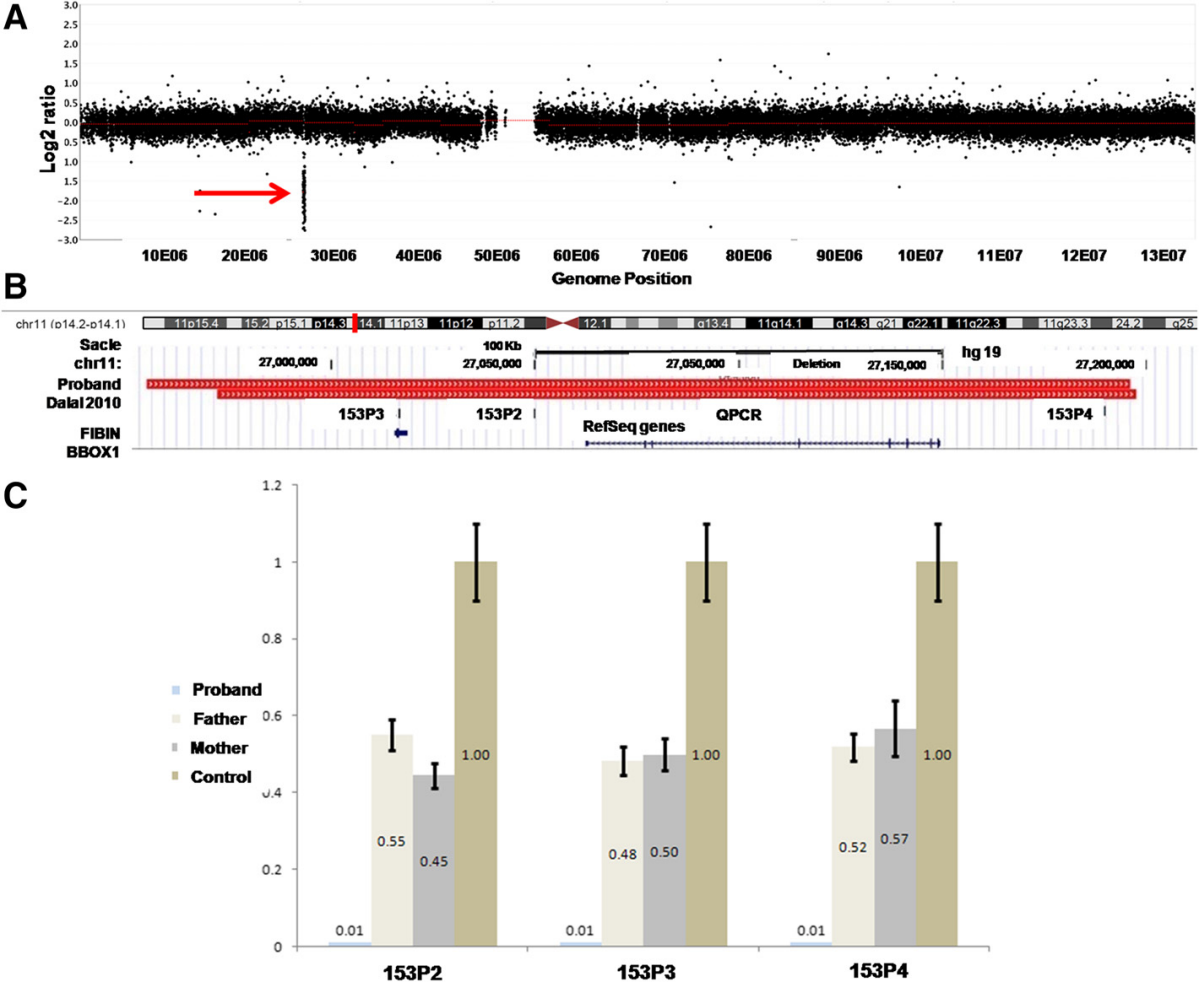
**Proband’s chromosome 11 genotype (A) NimblegenHuman CGH 3 × 720 K Whole-Genome Tiling array profile of proband’s chromosomes 11.** The homozygously deleted segment is pinpointed by a red arrow **(B)** Chromosomal position (top, marked by red box) and extents (bottom, red rectangles) of the homozygote deletion identified in the proband (top) and the heterozygote deletion described in a patient affected by Primrose syndrome [17] (bottom) in the UCSC genome browser. Both rearrangements encompass the *Fibin* and *BBOX1* genes (GENCODE genes indicated in blue at the bottom of the panel [19]). The positions of the amplimers used to confirm the array CGH results are indicated by ticks (153P2-153P4). **(C)** Relative DNA amount was quantified by QPCR at human chromosome 11 loci defined in panel **(B)** for the proband, her mother and father, as well as an unaffected control. Data from three amplification reactions are represented as mean ± S.D.

The proband’s karyotype at a resolution of about 350 bands was normal. NimblegenHuman CGH 3 × 720 K Whole-Genome Tiling v3.1 Array analysis [13] of her genomic DNA showed a 241 Kb interstitial homozygous deletion at 11p14.2 (Figure 2A-B). The complete genotype of the proband is therefore arr 11p14.2 (26,954,789-27,196,089) × 0. We used QPCR as previously described [14] to confirm this rearrangement in the proband and to show that both parents are heterozygous for the deletion (Figure 2B-C). This region encompasses *Fibin* (Fin Bud Initiation Factor Homolog) and *BBOX1* (butyrobetaine-gamma 2-oxoglutarate dioxygenase 1) (Figure 2B), a TGF-beta dependent gene that encodes a secreted protein essential for pectoral fin bud initiation in zebrafish [15,16] and an essential component of *de novo* carnitine biosynthesis [5,10], respectively. A similar almost fully overlapping heterozygous 225 kb deletion (Figure 2B) was suggested to be the cause of Primrose syndrome (OMIM#259050) at the heterozygote state [17]. The absence of ossified ear cartilage and muscle wasting specific to this pathology in the proband and both her parents, who are homo- and heterozygote for this rearrangement, respectively, goes against the implication of this deletion in this syndrome. Corroboratingly, some patients described in DECIPHER inherited similar deletions from their unaffected parents [18].

The proband’s metabolic investigations were performed in dry blood spot using tandem mass spectrometry (MVZ wagnerstibbe für Laboratoriumsmedizin). The indices of common blood metabolites, electrolytes, liver function tests, creatine phosphokinase, lipid profile, blood gas and amino acid profiles were all in the normal range in particular that of acyl-carnitines and isovaleryl carnitine (Table 2). A detailed analysis of the different acylcarnitine levels at 42 and 60 months of age showed that all evaluated acylcarnitines were in reference ranges, whereas free carnitine (FC) (25 and 19 μmol/L) was close to the normal lower limit (20–70 μmol/L) (Table 3). The proband’s acylcarnitine (15.5 μmol/L in proband, normal range 4–28 μmol/L) to free carnitine (AC/FC) ratio was normal however (age-specific range 0.1-0.8) (Table 3).

**Table 2:** Proband’s metabolic investigations

			
FBS (P)	98 mg/dL	Sodium (P)	140 mEq/L
BUN (P)	11 mg/dL	Potassium (P)	3.8 mEq/L
Creatinine (P)	0.6 mg/dL	Chloride (P)	102 mEq/L
Cholesterol (P)	164 mg/dL	17-OH-Progesterone (DBS)	< 5 nmol/L
Triglyceride (P)	63 mg/dL	TSH (DBS)	< 4 mU/L
HDL (P)	49 mg/dL	Ketone (U)	Negative
LDL (P)	102 mg/dL	Amino-acid TLC (U)	Normal pattern
SGOT (P)	22 IU/L	Ferric Chloride Test (U)	Negative
SGPT (P)	18 IU/L	DNPH Test (U)	Negative
CPK (P)	167 IU/L	Na-Nitropruside Test (U)	Negative
Lactate (P)	7.8 mg/dL	Amino-acids (DBS) (TMS)	unremarkable
Ammonia (B)	44 mcg/dL	Acylcarnitines (DBS) (TMS)*	unremarkable*
Galactose (DBS)	< 18 mg/dL	Isovalerylcarnitine (DBS)	unremarkable
GALT (DBS)	> 20% act	Glutaric acid (DBS)	unremarkable
PH (VBG)	7.392	Citrulline (DBS)	unremarkable
PCO2 (VBG)	32.1 mmHg	Arginosuccinate (DBS)	unremarkable
HCO3 (VBG)	19 mmol/L	Succinylacetone (DBS)	<5 μmol/L
BE (VBG)	-3.4 mmol/L	Biotinidase (DBS)	>40%act

*The detailed acylcarnitines results are further detailed in Table 3.

P: Plasma, B: Blood, U: Urine, FBS: Fasting Blood Sugar, BUN: Blood Urea Nitrogen, HDL: High Density Lipoprotein, LDL: Low Density Lipoprotein, SGOT: Serum Glutamic Oxaloacetic Transaminase, SGPT: Serum Glutamic Pyruvic Transaminase, CPK: Creatine Phosphokinase, GALT: Galactose-1-phosphate uridylyltransferase, VBG: venous Blood Gas, BE: Base Excess, TSH: Thyroid Stimulating Hormone, TLC: Thin Layer Chromatography, DNPH: Dinitrophenyl Hydrazine, DBS: Dry Blood Spot, TMS: Tandem Mass Spectroscopy.

**Table 3:** Proband’s carnitine and acylcarnitine profile

**Acyl carnitine**	**Quantity**^**1st **^**(μmol/L)**	**Quantity**^**2nd**^** (μmol/L)**	**Acyl carnitine**	**Quantity**^**1st **^**(μmol/L)**	**Quantity**^**2nd **^**(μmol/L)**
C0 (FC)	25.00	19	C12	0.13	0.17
C2	15.30	6.5	C12:1	0.04	0.12
C3	0.73	0.2	C14	0.08	0.03
C3-DC	0.04	0.04	C14:1	0.05	0.03
C4	0.63	0.46	C14:2	0.02	0.03
C4-DC	1.10	0.41	C14-OH	0.01	0.02
C5	0.10	0.07	C16	0.65	0.41
C5-DC	0.06	0.04	C16:1	0.06	0.03
C5-OH	0.17	0.1	C16-OH	0.01	0.01
C5:1	0.06	0.04	C18	0.42	0.26
C6	0.26	0.18	C18:1	0.59	0.28
C6-DC	0.00	0	C18:1-OH	0.01	0.01
C8	0.10	0.07	C18:2	0.16	0.07
C10	0.16	0.16	C18:2-OH	0.01	0.01
C10:1	0.15	0.09	AC/FC	0.84	0.52

1^st^: Measurements were done after 12 hours fasting at 42 months of age.

2^nd^: Measurements were done after 12 hours fasting at 60 months of age.

## Discussion

Carnitine is a critical molecule in the transport of fatty acids to the mitochondria and their subsequent beta-oxidation. Its homeostasis is maintained through oral intake, renal reabsorption and *de novo* biosynthesis. Although, the importance of carnitine dietary intake and renal reabsorption were thoroughly studied [9,20-23], the impact of *de novo* biosynthesis remains unclear as we lack animal models and a single patient deficient in enzymes involved in the carnitine biosynthesis pathway was described [8,11,12].

The female patient described here was referred to our genetic counseling and cytogenetic service because of microcephaly, dysmorphic features and IUGR. Array CGH revealed a homozygous deletion within chromosomal band 11p14.2 that deletes both the *Fibin* and the *BBOX1* genes, probably resulting in absence of *de novo* carnitine biosynthesis. The evaluation of metabolites involved directly or indirectly in the fatty acid b-oxidation pathway showed an increased ratio of AC/FC in the proband at 42 but not at 60 months of age (Table 3). Previous studies showed that such an increased ratio is an indicator of carnitine insufficiency; a situation in which there is inadequate free carnitine in response to an increase in metabolic needs [24-26]. It was further postulated that the AC/FC ratio reflects the intramitochondrial acyl-CoA/CoA ratio and thus that its alteration could be indicative of mitochondrial dysfunction [4]. The clinical and metabolic findings in the proband are not compatible with the described carnitine deficiency disorders [27-32] even if her levels of free carnitine were close to the lower limit of normal range demonstrating that the hypothesis that a defect in carnitine biosynthesis will not manifest itself as a systemic carnitine deficiency in omnivorous humans [8]. Corroboratingly, the TMLHE-deficient patients showed normal plasma carnitine level [11,12].

It is unclear if the clinical features shown by the proband, such as microcephaly for example, are associated with her minor carnitine insufficiency. The etiology of the different observed phenotypes could be associated to the absence of the *Fibin* gene, the second gene encompassed within the 11p14.2 deletion. Fibin is a secreted protein identified in zebrafish, mice and humans potentially acting downstream of retinoic acid and wnt signaling. It is essential for pectoral fin bud initiation and *tbx5* expression in zebrafish [16]. Alternatively they could be triggered by the reported perturbation of expression of copy-normal genes that neighbor structural rearrangement [33-37] or an unidentified recessive mutation inherited from both of her parents. Further studies are required to discriminate these different possibilities. Of note, consanguineous marriage is common practice and increasing in frequency in Iran [38,39]. It was suggested, however, that carnitine biosynthesis may be a risk factor for nondysmorphic autism as the TMLHE-deficient patient was identified in an autism spectrum disorder (ASD) cohort [11,12]. While our patient showed some dysmorphisms and global developmental delay she did not present autistic traits. Correspondingly she was microcephalic, a feature generally associated with schizophrenia rather than ASD that is generally associated with the mirroring macrocephaly [40-42].

## Conclusion

In conclusion, we present to our knowledge, the first patient with homozygous deletion of *BBOX1*, the second individual with a complete defect in carnitine *de novo* biosynthesis. She presents a mild decrease in free carnitine level but no clinical manifestations of carnitine deficiency disorders, suggesting that dietary carnitine intake and renal reabsorption are sufficient for carnitine homeostasis in omnivorous individuals.

## Consent

All samples used in this study were collected with the approval of the local ethics committee (“Commission cantonale vaudoise d'éthique de la recherché sur l'être humain”). Written informed consent was obtained from the patient’s parents for publication of this Case report and any accompanying images. A copy of the written consent is available for review by the Editor of this journal and appropriate informed consent. The latter signed by the parents includes the permission to publish pictures of the proband.

## Competing interests

The authors declare no competing interests.

## Authors’ contributions

ARN, SMA and AR designed the study. ARN, ST and HS prepared the necessary materials and produced the data. ARN and AR wrote the manuscript. All authors read and approved the final manuscript.

## Pre-publication history

The pre-publication history for this paper can be accessed here:

http://www.biomedcentral.com/1471-2350/15/75/prepub
